# Assessment of the effectiveness of PMTCT program in eight service delivery points in North Central Nigeria

**DOI:** 10.2147/HIV.S157685

**Published:** 2018-11-20

**Authors:** Obinna Ositadimma Oleribe, Ede Enenche, Deborah Udofia, Ekei Ekom, Princess Ifunanya Osita-Oleribe, Jin Un Kim, Simon David Taylor-Robinson

**Affiliations:** 1 Excellence and Friends Management Care Centre (EFMC), Abuja, Nigeria, obinna.oleribe@expertmanagers.org; 2 Hepatology Unit, Imperial College London, London, UK

**Keywords:** antiretroviral therapy, infectious diseases, prevention of mother-to-child transmission, women, Africa

## Abstract

**Background:**

Mother-to-child transmission (MTCT) of HIV is one of the commonest avenues through which infants are infected with HIV. To achieve an HIV-free generation, MTCT of HIV should be eliminated. Nigeria began prevention of mother-to-child transmission (PMTCT) services 13 years ago, but it still contributes to over one-third of global MTCT burden. We set out to explore and define the effectiveness of PMTCT in selected sites in North Central Nigeria.

**Methods:**

We conducted a retrospective secondary data analysis at eight service delivery points in two states. One thousand four hundred and fifty-four mother–infant pair data sets from 2012 to 2016 were extracted and analyzed. Maternal/infant antiretroviral (ARV) services, early infant diagnosis (EID), and final outcomes were reviewed to examine the predictors of MTCT of HIV in these centers.

**Results:**

We retrieved 1,454 mother–infant pair data sets. While 89.5% (1,302) of positive pregnant women (PPW) and 92.2% (1,340) of HIV-exposed infants (HEIs) received ARV prophylaxis/ARV treatment (ART), 88.4% (1,285) infants were breastfed with 32.5% still receiving breast milk at the time of dry blood spot (DBS) collection. EID PCR positivity rate was 3.5% (range, 0.0%–11.1%). Facility of delivery (χ^2^=24.99, *P*<0.00), mother on ARV (χ^2^=48.8, *P*<0.00), mother having received ARV prophylaxis (χ^2^=89.59, *P*<0.00), infant having received ARV prophylaxis (χ^2^=58.56, *P*<0.00), and baby having received cotrimoxazole (χ^2^=55.24, *P*<0.00) all significantly prevented positive EID results. However, mode of delivery and breastfeeding were not significantly associated with positive EID results.

**Conclusion:**

This study supports PMTCT services as it minimizes the transfer of HIV from infected mothers to HEIs. To eliminate HIV and achieve zero new HIV infections, every HIV-positive pregnant woman should receive ARV prophylaxis and should be supported postdelivery to prevent transfer of infection to the newborn. Also, HEIs should receive timely ARV and cotrimoxazole prophylaxis.

## Background

HIV has remained a major public health challenge, witĥ36.7 (range, 34.0–39.8) million people living with HIV, 1.8 (range, 1.6–2.1) million new infections, and 1 million mortalities by the end of 2016.^1^ To date, over 35 million lives have been lost to HIV and associated infections globally, with sub-Saharan Africa remaining the most affected region, as it accounts for two-thirds of the global total of new HIV infections and with 36.7 million people living with HIV in 2016.^1^ In Nigeria, over 3.1 million people are living with HIV, and the current national HIV prevalence is 3.0%, according to the recent national sentinel studies, among pregnant women attending antenatal care.^2^

HIV-positive woman can infect their babies during pregnancy, childbirth, and/or breastfeeding, and this accounts for >90% of new HIV infections among children.^3^ For instance, in the absence of any interventions during these stages, infected mothers can transmit the virus to their babies in 15%–45% of cases.^4^ In 2015, there were about 1.8 million children (age, 0–14 years) infected with HIV with 490,000 in West and Central Africa and 260,000 in Nigeria alone.^5^,^6^

Interventions to reduce mother-to-child transmission (MTCT) primarily involve antiretroviral (ARV) treatment (ART) for the mother and a short course of ARV drugs for the baby, measures to prevent HIV acquisition in the pregnant woman, and appropriate breastfeeding practices.^4^ Effective prevention of mother-to-child transmission (PMTCT) services require women–infant pair to have access to all relevant interventions.^7^ In September 2015, the WHO released a new guideline that recommended lifelong ART for all pregnant and breastfeeding women living with HIV, commonly called Option B^+^.^8^,^9^

PMTCT of HIV program began in Nigeria in December 2000 with the inauguration of the PMTCT National Task Team (NTT), while actual PMTCT services commenced as a pilot project in July 2002. This is in line with the WHO four-pronged approach.^10^ However, since inception, no structured evaluation of the effectiveness of the PMTCT services in Nigeria has been undertaken. Therefore, there is a paucity of data on PMTCT effectiveness of PMTCT programs.^11^ The purpose of this study was to determine the effectiveness of PMTCT program in selected sites in Nigeria.

## Methods

**Figure 1:**
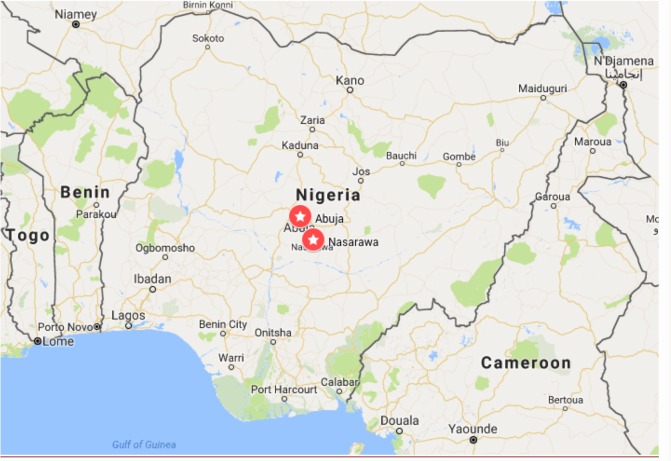
Map of Nigeria showing study locations – Nasarawa and Abuja, FCT. **Abbreviation:** FCT, Federal Capital Territory.

We purposefully selected eight supported sites in two states (Nasarawa and Abuja, Federal Capital Territory [FCT]; Figure 1). This study was conducted in sites supported by Excellence and Friends Management Care Center (EFMC), a nongovernmental organization involved in comprehensive HIV health care delivery across a wide range of Nigerian states.

Mothers whose infant details were documented in the registers sufficient for analysis and mothers in care for >6 months were included in this study.

We designed a data extraction form (DEF) using Micro-soft Excel spreadsheet to extract and record data. Ethical approval was obtained from the Nigerian Institute of Medical Research (IRB/16/354). All data collected were anonymized to remove identifiers and analyzed in aggregates; therefore, patient consent was not required. The training day included review of the data extraction template, data collection processes, use of Excel sheet, and review of entries. Data collection took place between December 2016 and January 2017. One thousand four hundred and fifty-four data sets were extracted from the early infant diagnosis (EID) PCR request and result forms and the child follow-up register across the eight facilities in the two states. Additional information was obtained from Delivery and Maternal registers. The chi-squared test was utilized to test for significance, and data were validated and analyzed using MS Excel, SPSS version 23, and OpenEpi.^12^,^13^

## Results

A total of 1,454 mother–infant data sets were extracted from the eight health care facilities (seven public and one private) located in Abuja, FCT (7; 87.5%) and Nasarawa (n=1, 12.5%). There were four primary and four secondary level facilities (Table 1), but 1,207 (82.8%) of the extracted data were from secondary level of care.

There were 50.8% female infants (n=738) and 48.3% male infants (n=703), with 0.9% missing gender data (n=13). The average age of the 1,453 (99.9%) infants whose ages were properly included in the data set at the time of dry blood spot (DBS) collection was 11.2±18.1 weeks with a median and mode of 7 and 6 weeks, respectively. Maternal age range was 18–40 years.

One thousand four hundred and forty-one (99.1%) had a documented reason for DBS, with 99.9% (1,440) being the first test for the healthy exposed baby. One thousand three hundred and two (91.0%) mothers were on ARV services. Although 1,166 (93.8%) positive pregnant women (PPW) received ART, only 445 (37.5%) PPW started their ART during the index pregnancy, and 1,118 (76.9%) were placed on triple regimen (Table 2).

One thousand three hundred and forty (96.5%) infants received ARV prophylaxis for HIV, 1,285 (92.2%) were breastfed, and 89.1% were breastfed exclusively. Extracted data showed that among those with relevant details, 85.4% were still on breast milk at the time of DBS collection and 91.3% received cotrimoxazole prophylaxis (Table 3).

The first EID HIV positivity rate was 3.5% as 47 of 1,339 infants tested positive to HIV, and the second EID HIV positivity rate was 1% among the exposed. Positivity rate for first EID ranged from 0.0% in three facilities to 11.1% in one facility (χ^2^=24.99, *P*<0.00). Mothers who were on ART were statistically less likely to have HIV-infected infants (χ^2^=54.71, *P*<0.00). Also, infants of mothers who received ARV prophylaxis (χ^2^=97.49.59, *P*<0.00), infants who received ARV prophylaxis (χ^2^=67.44, *P*<0.00), infants who were exclusively breastfed (χ^2^=14.07, *P*<0.00), and infants who received cotrimoxazole prophylaxis (χ^2^=55.97, *P*<0.00) were statistically less likely to be HIV infected when compared to the rest (Table 4).

Most deliveries took place in the primary health care center (61.5%), followed by secondary facilities (23.1%) and maternity homes (14.9%). Others had their babies at their private homes (0.8%). The place of delivery (c^2^=1.49, *P*=0.68) was statistically not significantly related to EID results. From the total birth, 95.5% were delivered through spontaneous vaginal delivery (SVD). The mode of delivery (XY_ates_=6.62, *P*=0.01) was significantly associated with positive EID results.

The reasons for second EID included repeat test after cessation of breastfeeding (n=133, 85.8%) and repeat test to confirm the original result (n=12, 7.7%). Other reasons included first testing for the healthy exposed baby and followup for breastfeeding children (n=3, 0.9% each) and repeat testing because of technical problems with the first test (n=2, 1.3%). The rest included first testing for a sick baby and/or to confirm positive PCR.

In the final outcome results analysis, those who became sick or died increased from 1.3% to 2.7% between the 6 and 12 months postdelivery.

## Discussion

DBS for HIV PCR was performed on all infants, and in 99.9% of these infants, DBS was the first test for a healthy exposed baby. The rest were tests for sick babies. The high DBS performance rate is a pointer to quality PMTCT services, as all children from both booked and unbooked mothers were tested for HIV at delivery or soon afterward. Thus, the number of sick infants who were tested for the first time, simply because they were sick, was a very small proportion of the total. However, the majority of the repeat tests was needed after cessation of breastfeeding (85.8%) and to confirm the first PCR test (7.7%). This result may be skewed because of lost to follow-up of most infants and their infected mothers. However, this will need further studies for proper elucidation.

As mentioned in previous studies, ART services, ARV prophylaxis, exclusively breastfeeding, and cotrimoxazole prophylaxis protected the HIV-exposed infants (HEIs) from infection before, during, and after birth, and this is statistically significant.^3^ The calculated positivity rate from this study of 3.5% is well within the acceptable limits.^4^ This is similar to the finding from a South African study^11^ but lower than a similar Nigerian study in 15 supported sites in five states, where the positively rate was found to be 5.3%. Other reported studies from South Africa and Kenya recorded positivity rates of 8.8% and 10%, respectively.^14^,^15^

On an individual basis, some sites had no HIV-positive children due to high-quality PMTCT services, while two centers had 8.0% and 11.0% positivity rates, respectively. These positivity rates were far higher than the expected <5% positivity rate, and the difference between centers was statistically significant. Additional review showed that the majority of the HIV-positive cases were women who were “unbooked”. Booking refers to the initial registration for antenatal care and PMTCT services. Therefore, the majority came into the facility to deliver for the first time and did not receive ARV prophylaxis as a consequence.

Regarding breastfeeding, the latest WHO guidelines recommend the national authorities to promote one infant practice among mothers with HIV, either exclusive breastfeeding while ARV drugs are provided or avoiding all breast milk.^16^,^17^ The breastfeeding rate (92.2%) was higher than findings from other African studies,^11^ which may have been linked to cultural practices that support breastfeeding in Nigeria. However, PMTCT may have helped to improve exclusive breastfeeding to 89.1% and cotrimoxazole prophylaxis to 91.3% among the surveyed community against 17% and 13% exclusive breastfeeding rate in 2013 and 2008, respectively.^16^,^17^ Studies have also shown that environmental factors such as access to piped water, electricity, gas, and paraffin for fuel, limit the implementation of the WHO/United Nations Children’s Fund guidelines.^18^ This results in inappropriate infant-feeding choices such as mixed feeding and lower infant HIV-free survival.^18^

While the place of delivery was not statistically significantly associated with MTCT, the mode of delivery (cesarean section vs SVD) was statistically significant (*P*<0.00). Improved tracking of HEIs is needed in PMTCT programs, where access to EID is still limited^19^ as the lowest prevalence of infant HIV infection or death was observed to occur among children completing the cascade.^20^

In this secondary data analysis, adherence, economic costs, the mothers’ behavior during highly active antiretroviral treatment (HAART), and the child feeding program during the therapy were not evaluated to ascertain how they could have affected the overall efficacy of ARV services in PMTCT.^21^ These are possible issues to be explored in future studies. Finally, one of the strengths of this study is that the result is free of self-reporting bias (including social desirability bias) seen in other studies where women were asked to self-report their ART use.^11^

Despite the effectiveness of PMTCT services, pediatric HIV infection is still common in Nigeria with the country contributing >30% to the global burden of the disease. Several nations in North America and Europe including Cuba have virtually eliminated pediatric AIDS,^21^ but to achieve this in Nigeria, there is the need to periodically assess the effectiveness of the PMTCT program and, based on the findings, modify the strategies and processes in place.

To accelerate PMTCT uptake and ensure that no woman or child is left behind, health care workers should initiate and facilitate provider-initiated and sustained patient counseling (PISC). PISC has five basic components: 1) every pregnant woman should be tested for HIV (and given their results); 2) PPW should be placed on ART or ARV prophylaxis once they are identified during pregnancy or in labor; 3) HEIs should be placed on ARV prophylaxis immediately after birth or at first appearance at a health care facility; 4) all HEIs should receive cotrimoxazole prophylaxis as long as it is required; and 5) HEI should not be breastfed if possible. But if breastfeeding is necessary, exclusive breastfeeding should be encouraged.

## Limitations

PMTCT services in Nigeria are fraught with multiple challenges. In this study, there were missing information from incomplete documentation, lost to follow-up, and delay in result delivery to the facility and to caregivers. Dates were not properly entered and thus were difficult to analyze. This is not unexpected as previous studies have documented the various difficulties encountered (including personnel and infrastructure requirements) with implementing PMTCT and EID services in resource-limited settings, such as Nigeria, which make proper monitored, effective counseling and quality service delivery difficult, if not impossible.^16^,^17^ Thus, despite the substantial benefits of PMTCT and EID to HIV-infected and HIV-uninfected infants, their families, and programs providing PMTCT services, access to quality services is hindered by these limitations.^16^,^17^ Only eight sites in two states were analyzed for this study, and therefore, the results are not representative of Nigeria. Although the findings are instructive and should guide programming, there is a need for a wider national survey using nationally representative samples. There will also be the need to compare implementing partners’ results, states, and facilities outcomes to identify key challenges and barriers toward eliminating MTCT of HIV in Nigeria.

## Conclusion

Although services have been fully decentralized in Nigeria to primary health care facilities, large number of clients (82.2%) still visits the secondary-level facilities, as shown in this study. Improving infrastructures and human resources at the secondary level of care will facilitate the provision of quality and sustainable PMTCT services to pregnant women and supervisory support to linked PHCs. This will culminate in better outcome for all HIV-PPW and HEIs.

To ensure MTCT of HIV is eliminated and impact on the infant minimized, there is a need to make greater efforts to reach all mothers, provide care to all PPW, and screen all HEIs early enough to provide care and treatment to the infected individuals.

## Figures and Tables

**Table 1:** Data extraction details from study facilities in Abuja and Nasarawa

Facilities	Level of care	No extracted	Frequency (%)
A	Secondary	116	8.0
B	Primary	53	3.6
C	Primary	17	1.2
D	Primary	51	3.5
E	Secondary	264	18.1
F	Secondary	100	6.9
G	Secondary	727	50.0
H	Primary	126	8.7
Total		1,454	100.0

**Table 2:** DBS collection and maternal ART details of PMTCT clients in study facilities

	Frequency	Percentage

**DBS**		
Reason for DBS		
First test for healthy exposed baby	1,440	99.9
First test for a sick baby	1	0.1

Total	1,441	100.0

**Mother on ART**		
Yes	1,302	91.0
No	67	4.7
Unknown	62	4.3

Total	1,431	100.0

**Time when ART was commenced**		
Description		
ART started before pregnancy	357	30.1
ART started during pregnancy	445	37.5
ART started after pregnancy	4	0.3
Unknown	380	32.1

Total	1,186	100.0

**Mother receiving ART**		
Yes	1,166	93.8
No	23	1.9
Unknown	54	4.3

Total	1,243	100.0

**ART regimen for mothers**		
AZT/3TC/sdNVP in labor	30	2.6
AZT/sdNVP in labor	2	0.2
Triple regimen	1,118	96.3
Triple regimen	1	0.1
Unknown	6	0.5
None	3	0.3

Total	1,160	100.00

**Abbreviations:** ART, antiretroviral treatment; DBS, dry blood spot; PMTCT, prevention of mother-to-child transmission; AZT, zidovudine; 3TC, lamivudine; sdNVP, single dose nevirapine.

**Table 3:** Infant breastfeeding, ARV, and cotrimoxazole prophylaxis pattern for exposed children in supported sites

Response	Frequency	Percentage

**Infant prophylaxis**		
Yes	1,340	96.5
No	40	2.9
Unknown	9	0.6

Total	1,389	100.0

**Infant breastfed**		
Yes	1,285	92.2
No	107	7.7
Unknown	1	0.1

Total	1,393	100.0

**Mode of breastfeeding**		
Exclusive feeding	1,133	89.1
Mixed feeding	139	10.9

Total	1,272	100.0

**Still breastfeeding at the time of DBS**		
Yes	472	85.4
No	42	7.6
Unknown	39	7.0

Total	553	100.0

**Infant cotrimoxazole for prophylaxis**		
Yes	1,092	93.1
No	28	2.4
Unknown	53	4.5

Total	1,173	100.0

**Abbreviations:** ARV, antiretroviral drug; DBS, dry blood spot.

**Table 4:** Cross tabulation of EID results with various independent variables

	Yes	No	Unknown	Total	Chi square
**EID vs mother on ART**					
HIV negative	1180	51	48	1279	54.71; *P*<0.00
HIV positive	27	11	6	44	
Total	1207	62	54	1323	
**EID and mother received ART prophylaxis**					
HIV negative	1061	14	39	1114	97.49; *P*<0.00
HIV positive	24	8	7	39	
Total	1085	22	46	1153	
**EID and infant on ART prophylaxis**					
HIV negative	1216	30	7	1253	67.44; *P*<0.00
HIV positive	28	9	2	39	
Total	1244	39	9	1292	
**EID and breastfeeding**					
HIV negative	1158	98	1	1257	0.04; *P*<0.98
HIV Positive	37	3	0	40	
Total	1195	101	1	1297	
		**Exclusive breast feeding**	**Mixed feeding**	**Total**	
**EID and mode of breastfeeding**					
HIV negative		1025	121	1146	14.07; *P*<0.00
HIV positive		25	11	36	XYates=12.13; *P*<0.00
Total		1050	132	1182	
		**Normal**	**C/S**	**Total**	
**EID and mode of delivery**					
HIV negative		55	2	57	6.62; *P*=0.01
HIV positive Total		1	0	1	
		56	2	58	
	**Yes**	**No**	**Unknown**	**Total**	
**EID and baby on cotrimoxazole prophylaxi***s*					
HIV negative	999	20	48	1067	55.97; *P*<0.00
HIV positive	22	7	1	30	
Total	1021	27	49	1097	
	**Home**	**Maternity home**	**Primary facility**	**Secondary facility**	
**EID and place of deliver***y*					
HIV Negative	1	17	73	26	1.49; *P*=0.68
HIV Positive	0	1	4	0	
Total	1	18	77	26	
**EID and facilit**y	**Chi Square 24.99; *P*<0.00**				

**Abbreviations:** ART, antiretroviral treatment; EID, early infant diagnosis.
